# Reliability of the triangle completion test in the real-world and in virtual reality

**DOI:** 10.3389/fnhum.2022.945953

**Published:** 2022-08-12

**Authors:** Ruth McLaren, Shikha Chaudhary, Usman Rashid, Shobika Ravindran, Denise Taylor

**Affiliations:** Rehabilitation Innovation Centre, Health Research and Rehabilitation Institute, School of Clinical Sciences, Auckland University of Technology, Auckland, New Zealand

**Keywords:** spatial navigation, virtual reality, triangle completion test, reliability, navigation, wayfinding, spatial cognition, vestibular

## Abstract

**Background:**

The triangle completion test has been used to assess egocentric wayfinding for decades, yet there is little information on its reliability. We developed a virtual reality (VR) based test and investigated whether either test of spatial navigation was reliable.

**Objective:**

To examine test-retest reliability of the real-world and VR triangle completion tests. A secondary objective was to examine the usability of the VR based test.

**Materials and methods:**

Thirty healthy adults aged 18–45 years were recruited to this block randomized study. Participants completed two sessions of triangle completion tests in the real-world and VR on the same day with a break between sessions.

**Results:**

In both test versions distance from the endpoint and angle of deviation showed poor test-retest reliability (r < 0.5). Distance traveled had moderate reliability in both the real-world and VR tests (*r* = 0.55 95% CI [0.23, 0.76]; *r* = 0.66 95% CI [0.4, 0.83, respectively]). The VR triangle test showed poor correlation with the real-world test.

**Conclusion:**

The triangle completion test has poor test-retest reliability and demonstrates poor concurrent validity between the real-world and VR. Nevertheless, it was feasible to translate a real-world test of spatial navigation into VR. VR provides opportunities for development of clinically relevant spatial navigation tests in the future.

## Introduction

Autonomous spatial navigation is a complex cognitive skill fundamental to independence (Cogné et al., 2017; Colombo et al., 2017) and can be defined as the process by which we use cues to determine and travel the route to a goal (Brodbeck and Tanninen, 2012). Spatial navigation relies on the storage and retrieval of information within the brain to remember where a landmark is located and plan a route to this location, as well as using ongoing afferent information to provide feedback along the route (Anson et al., 2018; Rogge et al., 2021). The strategies used for spatial navigation have been divided into allocentric (reliant on external visual landmarks and environmental cues) and egocentric (person/self- centered frame of reference) strategies (Colombo et al., 2017). While individuals may show a preference for one method over the other, the ability to switch and combine strategies flexibly depending on the demands of a task is essential for success in both familiar and less familiar environments and has been called wayfinding (Colombo et al., 2017).

Age, vestibular dysfunction, and cognitive impairment have been associated with deterioration in spatial navigation abilities. Older adults demonstrate reduced allocentric abilities, difficulty switching between spatial navigation techniques and deterioration in spatial memory (Gazova et al., 2013; Popp et al., 2017; van der Ham and Claessen, 2020). While people with vestibular disorders present with impaired allocentric and egocentric navigation (Glasauer et al., 2002; Brandt et al., 2005; Xie et al., 2017), take longer to navigate, and make more turning errors (Péruch et al., 1999, 2005; Borel et al., 2004).

Although impaired spatial navigation is a disability for many people, currently there are insufficient robust spatial navigation tests validated for use in a clinical setting. Current testing involves seated pen and paper or two-dimensional computer-based tasks during which the vestibular system has minimal stimulation relevant to movement through space (Guilford, 1956; Money et al., 1965; Ekstrom et al., 1976; Vandenberg and Kuse, 1978; Moffat et al., 1998; Adamo et al., 2012; Pai et al., 2012; Chan et al., 2016; Wood et al., 2016). While these static tests can evaluate components of spatial navigation and spatial memory, their sedentary nature fails to test vestibular, and somatosensory cues, interaction with the environment, and the planning and cognitive resources required in real-world navigation (Brandt and Dieterich, 2016; Cogné et al., 2017; Colombo et al., 2017; Wei et al., 2020). The triangle completion test overcomes this lack of vestibular contribution and assesses components of spatial navigation not easily assessed in seated tasks.

The triangle completion test has been used as an assessment tool over the past 30 years (Loomis et al., 1993; Glasauer et al., 2002; Wolbers et al., 2007; Dordevic et al., 2017, 2020a; Xie et al., 2017; Anson et al., 2019; Committeri et al., 2020; Wei et al., 2020; Rogge et al., 2021). It involves blindfolded participants being led along two segments of a triangle before being asked to independently rotate and navigate their way to the location of the starting position utilizing spatial memory and egocentric navigation strategies (Adamo et al., 2012). It has demonstrated differences in wayfinding abilities between healthy individuals and those with vestibular disorders (Glasauer et al., 2002; Xie et al., 2017; Anson et al., 2019) and associations with cognitive tests of visuospatial ability, executive function and perceptual motor speed and episodic memory (Committeri et al., 2020; Wei et al., 2020). It is reported to be sensitive to subtle age-related changes in the integration of sensory information (Adamo et al., 2012) and to changes in spatial navigation ability following balance and orientation training (Dordevic et al., 2017). However, there have been concerns with regards to the large differences within and between subjects and to date the reliability and validity of this measure has not been established (Glasauer et al., 2002; Wolbers et al., 2007; Xie et al., 2017). It is also limited by the need to eliminate all visual cues during the test making it unsuitable for the very frail or unsteady.

Virtual reality (VR) has been proposed as a means of controlling visual cues during spatial navigation tests without eliminating them entirely (Adamo et al., 2012; Cogné et al., 2017; Dorado et al., 2019a,b; Commins et al., 2020; Biju et al., 2021; Pastel et al., 2022). Early virtual environments used conventional computer screens. Participants remained seated and controlled an avatar or cursor to navigate a two-dimensional environment on the screen (Adamo et al., 2012; Brandt and Dieterich, 2016; Cogné et al., 2017). However, without the afferent proprioceptive and vestibular information usually acquired from motion, people typically demonstrate inferior performance on two-dimensional computer simulated tests (Adamo et al., 2012; Dorado et al., 2019b). Immersive three-dimensional virtual environments enable people to walk in a virtual environment and have shown advantages over evaluation in the real world (Biju et al., 2021; Pastel et al., 2022). An immersive virtual world enables uninhibited motion whilst simultaneously allowing the assessor precise environmental control of the visual scene. This enables elaborate environments to be simulated and manipulated with ease (Cogné et al., 2017; Harris et al., 2019). However, there are also differences in the visual experience in VR and the real-world that may affect spatial navigation, and early simulated environments have been reported to cause motion sickness in some people (Kennedy et al., 1993; Park et al., 2006; Adamo et al., 2012; Harris et al., 2019; Oh and Son, 2022; Pastel et al., 2022).

The aims of this study were to examine the test-retest reliability of the VR triangle completion test and the real-world triangle completion test, and to measure the agreement between scores on the real-world and VR triangle completion test. If reliable, then measures of validity would be determined. Usability of the VR triangle completion test will be explored both quantitatively and qualitatively *via* semi-structured interviews.

## Materials and methods

**FIGURE 1:**
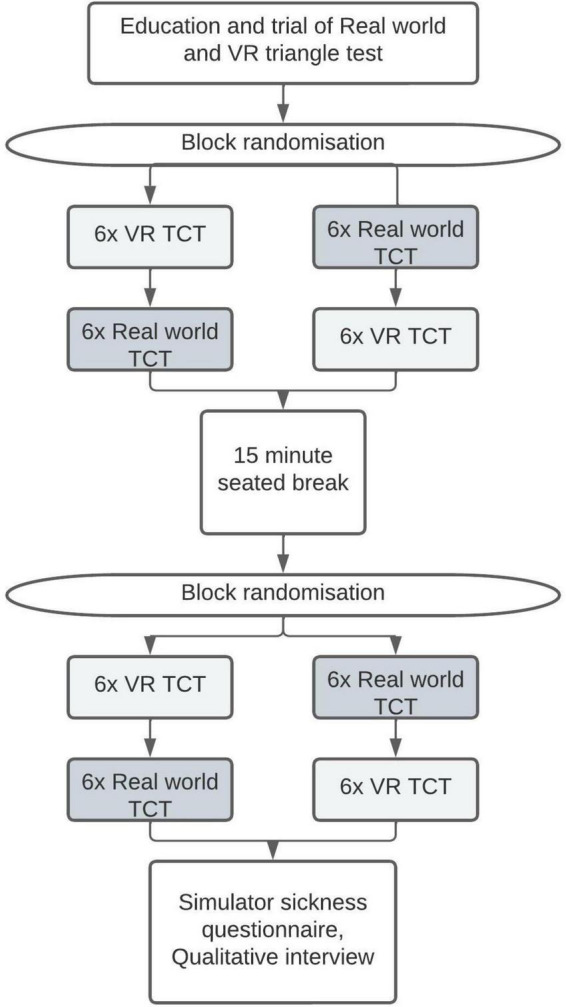
Flow chart of the study protocol. VR, virtual reality; TCT, triangle completion test.

This block randomized repeated measures study was designed to assess the test-retest reliability of the real-world and VR triangle completion test, and to evaluate the convergent validity of the VR triangle completion test against the real-world triangle completion test (Figure 1). Ethical approval for the study was given by Auckland University of Technology Ethics Committee (AUTEC) reference number 19/430.

### Participants

Thirty healthy adults aged between 18 and 45 years who were independently mobile with no history of neurological, vestibular, or balance disorder were recruited to the study from Auckland University of Technology, Faculty of Clinical Science staff and students.

### Study procedures

Participants were given training and practiced the real-world and VR tasks twice before the testing protocol began. The first practice trial in the real world was performed with the eyes open to enable familiarity with the task with the second practice done with vision obscured. In VR participants did both practice tests with the VR headset on. The first practice was to gain familiarity with the VR headset and the VR environment, the second test was to understand the test and respond to the visual instructions as part of the VR test. The task was practiced prior to data collection to reduce anxiety around testing that may affect performance and to eliminate the need for verbal instructions during testing that participants may unintentionally use as orientation cues. Then participants were block randomized to perform 6 repetitions of the triangle completion test in VR and 6 repetitions in the real-world. This was followed by a break of at least 15 min during which participants remained seated. They were then block randomized for a further 6 repetitions of the triangle completion test in VR and 6 repetitions in the real world. In total 12 repetitions of the triangle completion test were performed in VR and 12 in the real world. Each test was performed an equal number of times in the clockwise and anti-clockwise direction and an equal number of times with the shorter or longer leg of the triangle first in a random order (Figure 1).

Following completion of the VR testing, participants completed the simulator sickness questionnaire (Kennedy et al., 1993). This questionnaire was developed to quantify motion sickness in pilots undergoing flight simulator training. It contains a list of 16 common symptoms of motion sickness reflecting oculomotor, disorientation, and nausea components of visual-vestibular mismatch in the virtual world. The Simulator sickness questionnaire has been used as a descriptive measure to assess the usability of virtual reality applications involving both 2D and immersive environments (Kennedy et al., 1993; Park et al., 2006; Oh and Son, 2022). Each participant undertook a semi-structured interview comprised of open-ended and targeted questions. The format of questions was informed by a theoretical framework of acceptability for pilot and feasibility trials (Sekhon et al., 2017) and focused on the acceptability of the assessment and mode of delivery. The semi-structured interview started with open ended questions about participants opinions and experiences of the VR test, leading into targeted questions around physical issues, and ease of use (Hsieh and Shannon, 2005; Sekhon et al., 2017).

#### Real-world triangle completion test

**FIGURE 2:**
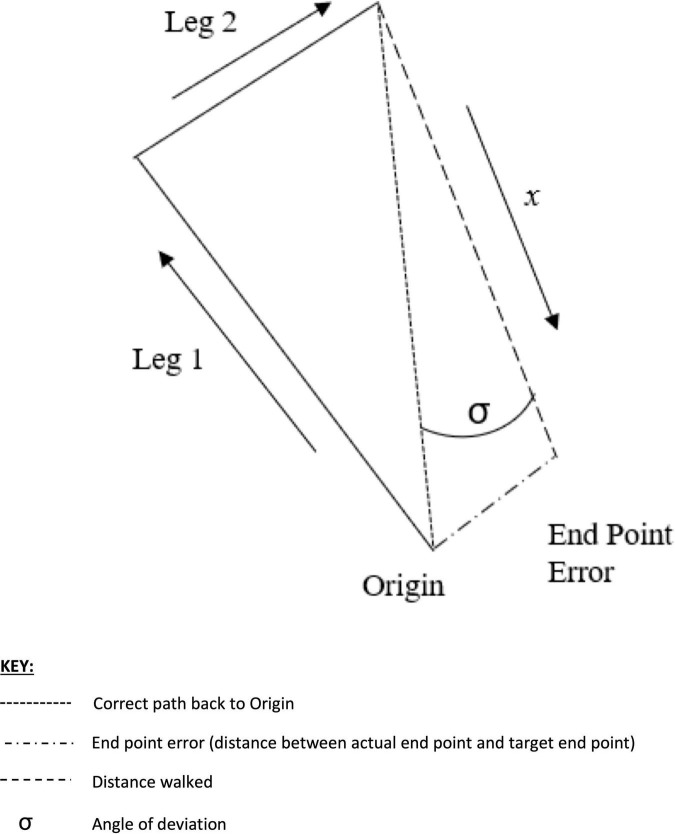
Visual representation of the triangle completion test.

In the real-world triangle completion test participants were asked to view one of two triangles (1 m × 2 m × 2.23 m with internal angles of 30, 60, and 90°) drawn on the floor of a room measuring 8 m × 7.4 m. The room used was part of a gait lab with clear floor area and devoid of extraneous furniture. Air conditioning units and any other machinery in the room that could emit unintentional auditory cues were switched off during testing. Participants stood at a corner orientated to approach the 90° angle first. They donned blackened goggles to obscure their vision and the assistant stood beside them and supported them around their shoulders. Participants were guided passively through the first segment of the triangle (1 or 2 m side). They were then guided through a 90° turn and along the next segment (1 or 2 m). The assistant then removed the guidance and asked the participant to independently turn and walk back along the hypotenuse to the start point. Once participants indicated that they were at their perceived start point a sticker was placed unobtrusively on the floor at the midpoint between the participant’s medial malleoli. Measurements were taken of the distance walked, the angle of deviation and the end point error using a digital goniometer and a tape measure once all 6 trials were completed (Figure 2).

#### Virtual reality triangle completion test

**FIGURE 3:**
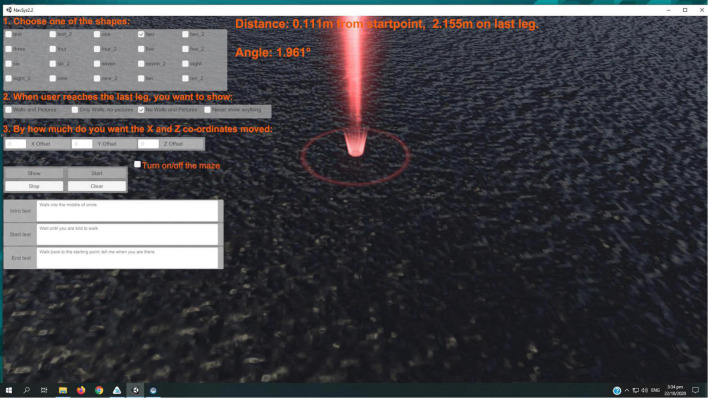
Image of the virtual environment.

The VR environment was developed iteratively by the team *via* a series of design workshops involving clinicians, patients, engineers, and software developers using Unity (C#). The participant donned a Vive Pro (HTC, Taiwan) VR headset and was orientated to a triangle shape on the floor (1 m × 2 m × 2.23 m with internal angles of 30, 60, and 90°). The virtual world was recreated in a 6.1 m × 3.8 m room designated specifically as a VR lab. The VR lab had unobstructed floor area and was devoid of extraneous furniture. Air conditioning units and any other machinery in the room that could emit unintentional auditory cues were switched off during testing. Participants were asked to walk to a beam of light indicating the start point and orientate their feet toward the 90-degree corner. When the test was ready to start the triangle and the beam of light disappeared and the participant walked to a beam of light that indicated where the corner of the triangle would be (Figure 3), turned 90°, then walked to a beam of light indicating the next corner. After walking the two segments the beams of light disappeared and a message appeared in front of them for several seconds instructing them to turn and walk back to the start point. Realtime 2D and 3D Unity interfaces tracked the movements of participants and recorded the distance walked, the angle of deviation and the end point error (Figure 2).

### Statistical analysis

Three outcomes were evaluated: distance from the end, absolute angle of deviation and total distance traveled. To evaluate differences across sessions, clockwise/anti-clockwise direction and 1 m/2 m length, linear and generalized liner mixed effects models were fitted to the data from the real-world triangle completion test and VR triangle completion test separately (R Core Team, 2013; Bates et al., 2015). Means and mean differences estimated from the models were reported along with their standard errors. Between-session test-retest reliability was assessed separately for the real-world triangle completion test and the VR triangle completion test. Reliability was evaluated for the mean of six tests from each session with Pearson’s product moment correlation (r). This coefficient is interpreted as the consistency of the instrument across two time points. If the reliability for an outcome was moderate or better, it was also assessed for the mean of the first four tests from each session. Convergent validity was only assessed for the reliable outcomes across the two tests. Convergence between the real-world and VR triangle completion tests was evaluated using Pearson’s product moment correlation. If the outcomes had moderate or better convergence, their absolute agreement was evaluated with a Bland–Altman plot. In addition to a qualitative assessment of the plot, the bias and limits of agreement were reported. The bias was interpreted as the systematic error between the two instruments. The limits of agreement were interpreted as the range of values which explain 95% of the differences in scores from the two instruments. Normality of the model residuals and outcome measures was evaluated with QQ-plots. Statistical significance threshold was set at 0.05. The magnitude of the correlation coefficients was interpreted as excellent (>0.900), good (0.750–0.899), moderate (0.500–0.749), and poor (<0.500) (Portney and Watkins, 2009).

Qualitative interviews were analyzed using direct content analysis (Krippendorff, 2018). Familiarization with the data was achieved by repeated reading. Initial coding was performed by identification of content related to usability. This data was then coded according to pre-determined themes of usability (ease of use and physical issues) and remaining data were examined for any further emerging themes. Text could be coded more than once if it provided a description of more than one concept (Hsieh and Shannon, 2005). Rank order comparisons of frequency of responses was used to determine ease of use and testing preference (Hsieh and Shannon, 2005). Pseudonyms were used in the reporting of results to maintain anonymity.

## Results

### Participants

Thirty participants were recruited to this study, 60% were female. The median age was 30 years (range 18–44, SD 7.28).

### Differences across sessions, direction, and length

In the real-world triangle completion test, the mean distance from the end was 49.2 cm ± SE 3.4 for session 1. There was a statistically significant (*z* = −2.2, *p* = 0.03) decrease (−5.8 ± SE 2.7) at session 2. The mean angle of deviation was 9.1° ± SE 1.1 for session 1 with no statistical differences (*p* > 0.05) across time periods. The mean distance traveled was 198.7 cm ± SE 3.8 for session 1. There was a statistically significant (*z* = 7.4, *p* < 0.0001) increase (15.9 ± SE 2.2) at session 2. There were no statistical differences (*p* > 0.05) across clockwise/anti-clockwise direction or 1 m/2 m length.

In the VR triangle completion test, the mean distance from the end was 46 cm ± SE 3.5 for session 1. There was a statistically significant (*z* = −2.4, *p* = 0.016) decrease (−6.8 ± SE 2.8) at session 2. The mean angle of deviation was 9.5° ± SE 1.1 for session 1. There was statistically significant (*z* = −2.6, *p* = 0.01) decrease (20% ± SE 1%) at session 2. The mean distance traveled was 220.5 cm ± SE 6.6 for session 1. There were no statistical differences (*p* > 0.05) across sessions. There were no statistical differences (*p* > 0.05) across clockwise/anti-clockwise direction or 1 m/2 m length.

### Test-retest reliability

#### Real-world triangle completion test

**FIGURE 4:**
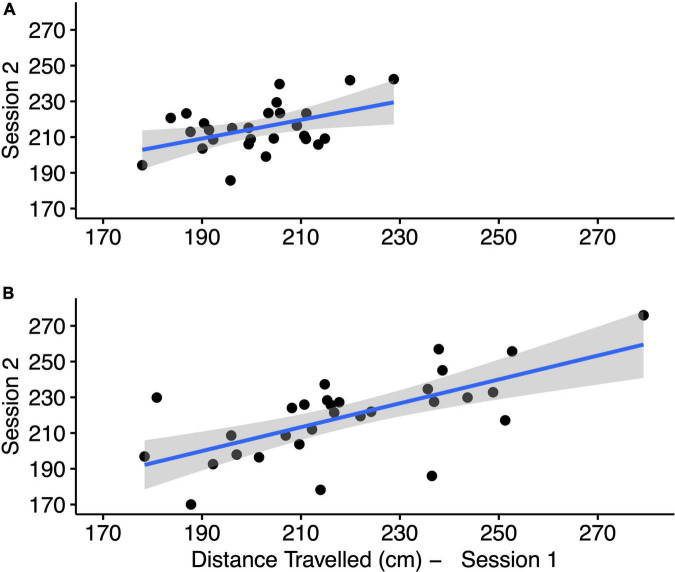
Raw data with least-squares regression line for the mean of six tests from session 1 vs. the mean of six tests from session 2 for the real-world triangle competition test **(A)** and the VR triangle competition test **(B)**.

Performing the real-world triangle completion test, using the average across six tests, distance from the end and angle of deviation showed poor test-retest reliability (r < 0.5). Only the distance traveled metric showed moderate reliability (*r* = 0.55 95% CI [0.23, 0.76]). The raw data from the two sessions is shown in Figure 4A. Using the first four tests, the reliability for distance traveled was also moderate (*r* = 0.53 95% CI [0.2, 0.75]).

#### Virtual reality triangle completion test

In the VR triangle completion test, using the average across six tests, distance from the end and angle of deviation showed poor test-retest reliability (r < 0.5). Only the distance traveled metric showed moderate reliability (*r* = 0.66 95% CI [0.4, 0.83]). The raw data from the two sessions is shown in Figure 4B. Using the first 4 tests, the reliability for distance traveled was also moderate (*r* = 0.65 95% CI [0.38, 0.82]).

### Convergent validity

#### Correlation between the real-world and the virtual reality triangle completion test

**FIGURE 5:**
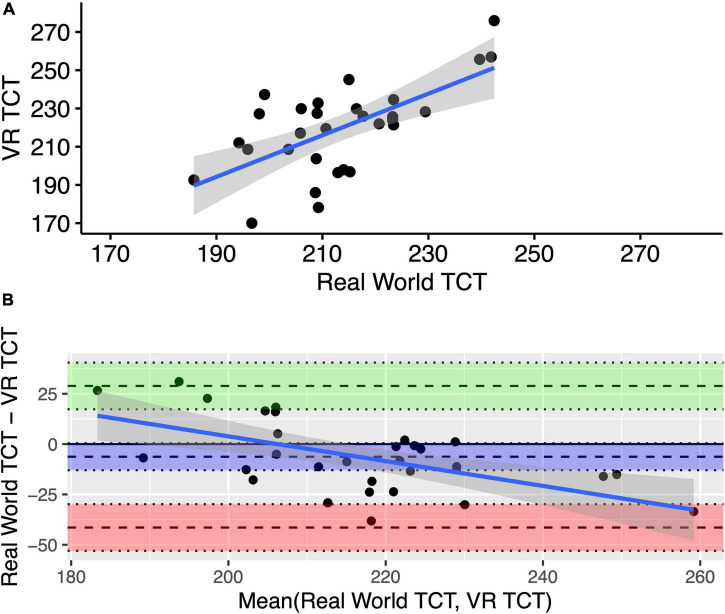
Raw data with least-squares regression line **(A)** and Bland–Altman plot **(B)** for the mean of six tests for distance traveled from the real-world and VR triangle competition tests from session 2. TCT stands for triangle completion test.

The VR triangle completion test showed poor correlation against the real-world test for distance traveled using the average across six tests from session 1 (*r* = 0.45 95% CI [0.09, 0.7]). The VR triangle completion test showed moderate correlation against the real-world test for distance traveled using the average across six tests from session 2 (*r* = 0.64 95% CI [0.37, 0.81]). The raw data for the average across six tests from session 2 for the two tests is shown in Figure 5A.

#### Absolute agreement between the real-world and the virtual reality triangle completion test

The Bland-Altman plot for the means of six real-world and six VR triangle completion tests for distance traveled during session 2 are shown in Figure 5B. The plot suggests that the systematic difference between the two instruments is not constant. While neither data set had a normal distribution, there was a greater spread of data in the VR test and more variance in the VR results compared to the real-world results. When participants walk longer than the prescribed distance, they walk a greater distance in VR compared to the real-world test. When participants walk shorter than the prescribed distance, they walk a shorter distance in VR compared to the real world. Without normal distribution of differences, the Bland-Altman bias and limits of agreement statistics do not have a valid interpretation. Thus, the two tests, VR and real-world have a poor absolute agreement with each other.

### Usability

The median total simulator sickness score was 15.45867 (SD 17.31337). When broken down into subscales the nausea sub score was 9.858 (SD 13.13406), oculomotor sub score was 13.58267 (SD 15.72199), and the disorientation sub score was 17.178 (SD 22.7664).

Qualitative content analysis found that 21/30 perceived the VR test was easier to use than the real-world test. The main reason given for finding it easier was the visual input from the VR that contributed to a perception of better balance and stability during the test. People also reported feeling more autonomous in VR as gait and trajectory was under their own volition as opposed to being guided through the path by the researcher. Eight participants felt that the VR test was more difficult than the real-world test. The main reasons given for finding the VR test more difficult were related to the worded instructions coming up on the screen which people were either unable to read, found disappeared too quickly or contributed to eye strain and oculomotor fatigue. This was particularly noted in participants who routinely wore prescription glasses that were removed to use the VR headset. While it was not specifically asked, seven people volunteered that they perceived that the triangle completion test got easier with repetition with the main reason given or implied being familiarity with the testing process. Five people reported the test got harder with more repetition with one person reporting a growing sense of disorientation, and four reporting eye strain as the reason for this. All participants felt they were able to understand how to perform the test in VR. The verbal instructions from research staff were considered the most useful aspect of communication.

One aspect of usability that emerged as a theme during content analysis of the qualitative interviews was participant’s sense of presence in the VR environment. Overall, participants reported an excellent sense of presence and engagement in the environment.

“It was like stepping into a whole different world. So, you feel like you’re just transported to somewhere else. And, the actual graphic, I felt like I was, it’s solid but almost looks like water and so, was quite an interesting sensation to be in there with this quite empty void of a place.” (Emily, 20 year-old female)

However, there were a small number of people for whom their presence in the environment was impacted by the distraction of a residual sense of the real world preventing them from suspending reality entirely. The primary distractors were, a sense of disembodiment, visual disturbances when people had to remove prescription glasses to put the VR headset on and the distraction of the hardware (the weight of the headset or the battery and it’s cord).

During the interviews it became clear that VR as a means of assessment was acceptable to participants. Of the 24 people who were asked, 17 preferred the VR test, 5 the real-world and two had no preference. Participants described the VR test as “fun,” they “enjoyed it,” “got caught up in it,” and reported “it felt like a game.”

## Discussion

### Test-retest reliability of the triangle completion test

The triangle completion test is a simple to perform test of spatial navigation that on face value has the potential to assess contribution and ability of the vestibular system to orientation in space. Variations of the triangle completion test have been used in research over the past 30 years (Loomis et al., 1993; Glasauer et al., 2002; Wolbers et al., 2007; Dordevic et al., 2017, 2020a,b; Xie et al., 2017; Commins et al., 2020; Rogge et al., 2021). However, until now reliability has not been explored.

Our study is the first study to report poor test-retest reliability of the triangle completion test. This makes it inappropriate to use the triangle completion test as a clinical outcome measure in its current form, as a change in result cannot necessarily be attributed to real clinical change. While we are the first group to report reliability formally, caution with regards to the reliability and sensitivity of the test has been raised by numerous researchers in the past three decades. Loomis et al. (1993) questioned whether proprioceptive and vestibular cues were adequate for wayfinding and Xie et al. (2017) raised concerns about the sensitivity of the test. Glasauer et al. (2002) and Wolbers et al. (2007) highlighted large inter individual differences for all parameters of the triangle completion test. With Wolbers et al. (2007) noting considerable variation within subjects, that tended to be statistically obscured by taking the mean responses of the data, a tendency that can be noted in our results as well.

At a group level our results indicate research using the triangle completion test as a primary outcome measure must be interpreted with caution. While the significant difference between session one and session two was in the direction of improved accuracy for both real-world and VR tests the reliability remained poor with only the distance traveled demonstrating a moderate level of reliability. It seems likely that the change between sessions indicates a repetition effect, with participants learning from repeated exposure to a novel task. Most research has involved 4–6 repetitions of the TCT (Glasauer et al., 2002; Dordevic et al., 2017, 2020a,b; Xie et al., 2017; Anson et al., 2019; Wei et al., 2020; Rogge et al., 2021) whereas our participants completed 6 real-world and 6 VR tests in session one and repeated this in session two. It is possible that there was a cumulative learning effect between the two tests as the task requirements were the same, despite the change in environment. The moderate test-retest reliability for distance walked requires further investigation. Distance errors during the triangle completion test have been correlated with loss of vestibular function (Xie et al., 2017; Anson et al., 2019). However, in the absence of vision, distance walked can be influenced strongly by proprioceptive cues of step length and cognitive processes including counting steps (Glasauer et al., 2002). Therefore, it is unlikely that this metric is meaningful when taken alone.

Like Pastel et al. (2022), we found the angle of the final turn had no effect on end point accuracy in a healthy young adult population. In comparison, Adamo et al. (2012); Anson et al. (2019), and Cuturi et al. (2021) who investigated wayfinding in children and older adults, found that the turning angle did influence accuracy, with a larger angle of turn leading to reduced end point accuracy. This is worth investigating further as sensitivity to rotation may alter across the lifespan (van der Ham and Claessen, 2020). Alternatively, if the size of turning angle does not influence spatial navigational ability less repetition will be required in future spatial navigation tests as it will not need to be controlled for. Fewer repetitions would result in less time required for testing and may minimize any repetition effect.

Anson et al. (2019) used the same triangle protocol as we did in healthy older adults with and without vestibular disorders. As could be expected, our mean end point and angle of deviation error is less than their results in older adults and those with vestibular disorders. Xie et al. (2017) had similar findings to us, however, as they used a smaller triangle we can draw limited meaning from this, highlighting the need for consistency in protocols to enable comparisons between studies.

### Agreement between scores on the real-world and virtual reality triangle completion tests

The results of this study also raise important questions about the validity of transferring real-world tests into the virtual world and how the medium of virtual reality may influence spatial navigation. To date, the use of VR and its influence on spatial navigation remains largely unknown (Pastel et al., 2022). The lack of agreement between the VR and the real-world test in our study suggests that the transferability of results from virtual applications into real-world scenarios cannot be assumed (Biju et al., 2021; Pastel et al., 2022).

Preliminary research has identified subtle differences in the cognitive processing of sensorimotor tasks in VR compared to the real-world (Adamo et al., 2012; Harris et al., 2019; Pastel et al., 2022). This appears to be specific to the individual VR setup and the task. In a distance estimation task Feldstein et al. (2020) found no significant differences in estimates of distance in their VR setup compared to the real-world. However, once a motion component was added, judging road crossing safety with a vehicle bearing down on them, different factors influenced people’s spatial cognitive perception in VR compared to the real-world (Feldstein and Dyszak, 2020; Feldstein and Peli, 2020). While people demonstrated consistent responses to car color in both scenarios (Feldstein and Peli, 2020), they responded differently to car velocity in the different environments (Feldstein and Dyszak, 2020). These discrepancies do not preclude the transfer of testing into a virtual world, nevertheless, the different mediums are likely to affect performance, and therefore, the ecological validity of the virtual assessment. This highlights the importance of comparative studies to better understand this phenomenon.

Current hypotheses around why cognitive processing of the virtual world may be different include the nature of the virtual image, the sense of disembodiment and the use of different visual pathways. In VR the virtual image is created from a small optic array on a flat screen close to the eyes that sends information to the brain constructing a large three-dimensional world. Unlike the real-world where the visual location of objects is consistent with their spatial orientation VR is dependent on the brain creating a plausible visual illusion of space and location that has an unknown effect on spatial navigation. Current VR technology carries with it a sense of disembodiment. People are unable see their own body or interact with the virtual environment, this may affect spatial relationships with objects which provide cues for orientation. In the real-world vision is primarily processed *via* a ventral pathway concerned with perception and identification of visual inputs. VR appears to primarily use a dorsal pathway more commonly used to control action. It is unknown how this influences cognitive processing of the virtual environment (Harris et al., 2019).

### Usability of a virtual reality triangle completion test

One of the challenges of virtual reality affecting the sense of presence and enjoyment of the environment can be a sense of motion sensitivity or motion sickness (Pastel et al., 2022). The simulator sickness score was used to quantify this. While the scale has no normative values our scores are in line with those of participants that have tolerated the virtual world well during a spatial navigation task in other studies (Park et al., 2006; Oh and Son, 2022; Pastel et al., 2022). Coincident to this finding motion sickness did not emerge as a theme in our study.

“I am otherwise really motion sick, unless I’m the person that’s driving, like 100% I will throw up if it’s a long distance. So it was interesting that this could be an issue with the headset, but I didn’t have it.” (Laura 26 year-old female)

A sense of presence in a virtual environment and the sense of being transported to a new reality is considered an important feature of successful virtual worlds (Dorado et al., 2019a; Grassini et al., 2021). Despite the environment being designed to be a void with no visual stimulus most participants reported good sense of presence within the environment. The sense of autonomy and improved stability supports further investigation of use of virtual reality in groups with poor balance or for whom we may not wish to remove visual cues entirely.

### Limitations

A limitation of the current study was the number of repetitions of the test in short succession. Participants completed two sets of 12 repetitions of the test (6 VR, 6 real-world) with a 15 min break in between. It is possible that the 15 min break was not sufficient to enable washout of any learning effect. A further limitation was the large number of repetitions in a short timeframe having potential to reduce participants attention to the task. As executive function has been found to be related to spatial navigation on the Triangle completion test (Wei et al., 2020) it is possible that this influenced the high variability in results.

### Looking to the future

With the need for caution interpreting triangle completion test results identified by researchers for decades, it is pertinent that this test has continued to be used so extensively (Loomis et al., 1993; Glasauer et al., 2002; Wolbers et al., 2007; Dordevic et al., 2017, 2020a,b; Xie et al., 2017; Committeri et al., 2020; Rogge et al., 2021). This highlights the lack of feasible alternatives available. The triangle completion test meets a number of key requirements of spatial navigation testing, it uses active locomotion, provides vestibular and proprioceptive cues from translation and rotation in space, it is simple and quick to perform, uses readily available equipment, a moderate amount of space and has low technical requirements. It also involves wayfinding, a conceptually valid method of assessing spatial navigation (Adamo et al., 2012). However, despite its advantages, there remain questions with regards the Triangle completion tests ecological validity. Performance on the Triangle completion test has been significantly associated with measures of spatial memory, executive function, motor processing speed (Wei et al., 2020), and otolith function (Xie et al., 2017) in older adults. However, to our knowledge, links between test results and spatial navigation performance in the real-world have not yet been demonstrated. While showing some conceptual benefits over other tests, the Triangle completion test is a laboratory based test that investigates gait trajectory over a short distance with vision obscured. The extent to how this may relate to the complexity of real-world navigational scenarios requires further investigation.

Spatial navigation requires integration of afferent sensory systems (vestibular, visual, and proprioceptive) with spatial memory and cognitive domains of the central nervous system. While the vestibular system is considered one of the primary sensory organs contributing information on heading direction and place in space (Smith et al., 2015), it works within the context of the visual and proprioceptive systems (Glasauer et al., 2002). When we eliminate vision it is possible that we remove information that the brain has come to depend on to provide context to other sensory systems disturbing central processing and making wayfinding unreliable, even in a young healthy population.

While our study has demonstrated poor test- retest reliability of the Triangle completion test in both the real-world and in VR and we have raised concerns around the validity of these test findings. We hope these findings may contribute to development of a more useful test in the future. Virtual reality enables the development of tests that manipulate visual cues as opposed to eliminating them. Embracing this technology and its ability to both manipulate the environment and track real time movement in three-dimensional space has the potential to help us unlock some of the factors involved in human navigation. The human analogue of the Morris water maze is a test that adapted to VR may contribute to deeper understanding of human navigation (Laczó et al., 2009; Gazova et al., 2013). Performed in a blue curtained arena to eliminate uncontrolled visual feedback, visual orientation cues are projected onto the floor and walls of the arena. Manipulation of these cues enable us to test spatial navigation using both egocentric and allocentric strategies (Laczó et al., 2009; Gazova et al., 2013). A limitation of the human analogue of the Morris water maze that prevents it being more widely used is the extensive equipment required to set up the arena and provide environmental cues. Our research has demonstrated that it is feasible to successfully translate a spatial navigation task into virtual reality. Translation of tests like the human analogue of the Morris water maze into virtual reality have the potential to make them more accessible to researchers and clinicians alike. However, realizing that the brain processes spatial information differently in virtual reality (Adamo et al., 2012; Harris et al., 2019; Pastel et al., 2022) it is important to investigate this carefully and determine whether performance in the virtual world provides a valid representation of an individual’s capabilities during everyday navigational tasks.

## Conclusion

The triangle completion test showed poor test-retest reliability between session one and session two confirming the concerns voiced by researchers over the past three decades about reliability of the test. The test was successfully translated into virtual reality identifying possibilities for future development of spatial navigation tests. Due to the complexity of spatial navigation and the multiple sensory inputs and cognitive domains involved, the validity of spatial navigation tests in VR and how we assess this requires careful thought.

## Data availability statement

The raw data supporting the conclusions of this article will be made available by the authors, without undue reservation.

## Ethics statement

The studies involving human participants were reviewed and approved by Auckland University of Technology Ethics Committee (AUTEC), Auckland University of Technology. The patients/participants provided their written informed consent to participate in this study.

## Author contributions

DT conceived the original idea. DT and RM contributed to the design and implementation of the research. RM, SC, and SR carried out the experiments. UR performed the statistical analysis. RM wrote the manuscript with input from all authors.
